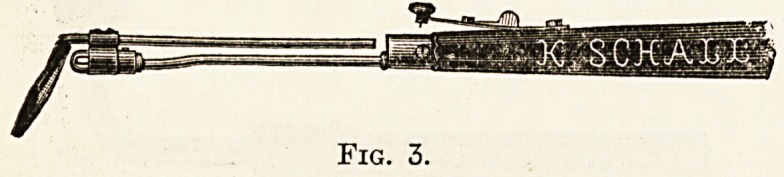# Apparatus for Light and Current from the Main

**Published:** 1912-01-13

**Authors:** Alfred C. Norman

**Affiliations:** House Surgeon at Sunderland and Durham County Eye Infirmary.


### ELECTRICITY IN MODERN MEDICINE.*

### V.—Apparatus for Light and Current from the Main.

By ALFRED C. NORMAN, M.D. Edin., House Surgeon at Sunderland and Durham County Eye Infirmary.

If *Leclanché Cells* are to be used for lighting small medical lamps, three of them must be connected in series to produce approximately four volts. Dry cells should be obtained; the principle is the same, but the exciting fluid is held in some absorbent material such as sawdust, and the whole thing is contained in a zinc box or cylinder, which also acts as the zinc plate of the cell. Cells of suitable size cost about 2s. 6d. each, but they cannot be re-charged when exhausted.

*Bichromate batteries* are heavy and dirty to use, but where one is already installed for cautery work it may be utilised for light as well. Two cells do not give quite four volts, and three cells give almost six volts, so it is best to use a battery of three or four cells and to control the current with a small variable resistance, such as fig. 1. This costs about 7s. 6d.

Since we require smaller currents and are dealing with larger voltages, it is necessary to use a much higher resistance than served for the cautery. If it were made of the thick wire that was used for the cautery-resistance, many yards would be required to produce the necessary number of ohms, and this would be too cumbersome for practical purposes; so a resistance for small lamps is made of fine wire wound round an insulating-rod. The one shown in fig. 1 can be used to control the current from any battery up to eight volts through any of the small voltage lamps used for medical purposes. When starting to use it the

Fig. 1.

sliding contact should be at "weak," and should be moved along gradually until the lamp gives the proper amount oı light.

The advantage of using metallic-filament lamps in medical instruments lies in the fact that they give as much light as the carbon ones from a considerably smaller current, and consequently do not become so hot.

The efficiency of an electric lamp is measured by the number of "watts" of current consumed in producing a candle-power of light. (To arrive at the number of "watts," the voltage of the supply must be multiplied by the number of ampères passing).

Carbon lamps consume between three and four watts per candle-power, whereas the more efficient of the new metallic-filament lamps require but one watt. By installing full voltage metallic-filament lamps in a house or hospital, in place of carbon ones, the bill for current can be reduced to less than one-half, but against this must be balanced the primary cost of the lamps (3s. 6d. as compared with 1s. for carbon ones), and the fact that they are rather fragile. The latest "drawn-wire" metallic lamps, however, promise to be as durable as the carbon ones, and they have the additional advantage of not becoming black, so that they can be used until burnt right out.

*Carbon* lamps *must* be used for charging accumulators on the house-mains. To find out what candle-power is required to let through a given ampèrage, it should be remembered that these lamps pass about 3.5 watts per candle-power; so we multiply the voltage of the supply by the number of ampères we want and divide the result by 3.5. For example, if an accumulator required a charging current of two ampères and the voltage of the supply happened to be 250, it would be necessary to use carbon lamps of 142 c.p., which could be attained approximately by putting three 50 c.p. lamps in parallel on the charging-board.

$$\frac{250 \text{ volts} \times 2 \text{ amperes}}{3{\cdot}5} = 142 \text{ c.p.}$$

In selecting an outfit of instruments for a small general hospital money would be well spent on the following: hand-lamp, cystoscope, head lamp, illuminated ophthalmoscope, and illuminated laryngoscope.
following: hand lamp, cystoscope, head lamp, with accessories for various purposes. With the

Fig. 2.

glass thimble *in situ* it may be placed in the mouth to trans-illuminate the maxillary antra. By putting on the bull's-eye lens we can obtain a concentrated light, which is useful in many deep operations, such as supra-pubic cystotomy. If the bull's-eye be partly covered over by the rubber guard the lamp may be used to trans-illuminate the frontal sinuses. For examining the tonsils and fauces a metal tongue depressor can be attached. The complete instrument costs about £2 10s.

*A head-band* with lamp and bull's-eye attached is often of great service when operations have to be done without an assistant. It costs about 35s.

*The cystoscope* should be found in every well-equipped hospital. Fenwick's pattern is excellent for general purposes. There is no need to purchase two instruments, as the "anterior" one can be used to see practically the whole interior of the bladder. Cystoscope lamps are small, expensive, and very fragile; they should always be used with a small variable resistance even on a battery of their own nominal voltage. The saving in lamps will soon repay the cost of the resistance. Fenwick's cystoscope costs about £5.

* Previous articles in this series have appeared in The Hospital of Nov. 11 and 25, Dec. 9 and 30.

The *illuminating attachment* invented by Dr. Marple for the *Morton ophthalmoscope* has revolutionised the method of directly examining the fundus oculi. There are many useless electric ophthalmoscopes on the market; the advantage of Dr. Marple's pattern lies in the shape of the mirror. It costs about £5 5s.

Fig. 3 shows the *electric laryngoscope* designed by Sir Felix Semon; it has many advantages and costs about 35s.

In using small lamps, with a resistance, they should never be allowed to give so much light that the outline of the filament cannot be seen. If a lamp fail to light up when the current is on, it may be that it is burnt out (in which case a break in the filament can be seen and the glass will be blackened), if this is not the case we should make sure that it is well screwed home and that the cords are properly connected. In some instruments the cords are permanently attached; in others the two poles, insulated from each other of course, are contained in a small concentric plug which has to be pushed into a hole in the handle. Care should be taken that the plug is not *forced* in obliquely or a short-circuit will result when the current is switched on.

There is a special attachment for cystoscope cords which allows of the instrument being rotated without disarranging the cords. A cystoscope should never be passed into the bladder with the current switched on, because the heat of the lamp might damage the urethra. Once it is in the bladder the

FIG. 3.

lamp will be kept sufficiently cool by the fluid, which is always introduced before a cystoscopic examination.

### CURRENT FROM THE MAIN.

So far we have considered electricity produced, or reproduced, as a result of chemical action; we now come to discuss current *induced* by mechanical means. The most efficient machine for converting mechanical into electrical energy is the *dynamo*; it is the prime source of electricity at all power stations, and may be driven by a steam or gas engine, or by water power.

The dynamo depends upon the principle that a current of electricity will be induced in a coil of wire if the coil be revolved in the presence of a magnet. In its simplest form the dynamo consists of *armature*, *field-magnets*, and *current-collecting mechanism*.

The *armature* consists of an axle, surrounded by strips of soft iron, upon which are wound many turns of insulated copper wire (the core of soft iron increases very markedly the efficiency of the dynamo). The axle, driven by a belt from an engine, runs in well-lubricated bearings placed so that the armature is revolved as close as possible to the " poles " of the field-magnets.

The *field-magnets* are electro-magnets so arranged that they are excited by the current (or a shunted portion of it) generated in the armature. There is usually enough residual magnetism in the field-magnets to start the inductive process in the armature, and once this has begun the magnets become exceedingly powerful.

The simplest form of *current-collecting mechanism* consists of two copper rings electrically insulated from each other and the axle, but attached to and revolving with the axle. The free ends of the armature winding are connected one to each of these rings, so that, when the armature is revolved by mechanical means, the current generated in its coils will be conveyed to the rings. Two stationary carbon collecting-brushes make contact with the revolving rings, and are connected with cables which convey the current to the switchboard and street mains.

The above description applies to an alternating-current dynamo. During one complete revolution of the armature, current is flowing in the street mains (and of course in the armature) for about one-half the time in one direction and for the other half in the opposite direction. The time which elapses between the *commencement* of a flow in one direction and the *commencement* of another flow in the same direction is known as a " period " or " cycle." In ordering electrical instruments for use with the alternating current it is essential to state the " periodicity " of the supply. The number of periods is obviously identical with the number of revolutions per second the dynamo at the power station is making. In this country the periodicity may be anything between 40 and 100 per second, but it is always constant for a given town or district.

In the majority of towns in England the current is rendered unidirectional or " continuous " before it enters the street mains. This is done by an arrangement on the collecting mechanism of the dynamo known as a " commutator," in which the collecting rings are replaced by many insulated copper segments, each connected to a portion of the armature winding. The carbon brushes thus collect current from each section of the armature at the same stage in its cycle, and so the current is rendered unidirectional in the external circuit.

The continuous supply is much more easily adapted to medical requirements than the alternating; but in certain large districts, where current has to be carried for long distances, it is necessary to use a voltage in the street mains so high that it would not be safe to apply the current for domestic lighting purposes. Now the alternating current possesses a great advantage over the continuous in that it is easily reduced to a lower voltage by means of a simple instrument known as an *alternating current transformer;* so in these districts it is usual to have an alternating current of high voltage in the street mains and to reduce it to a safe pressure by means of small transformers stationed at the points of entrance to the houses.

(*To be continued.*)

## Figures and Tables

**Fig. 1. f1:**
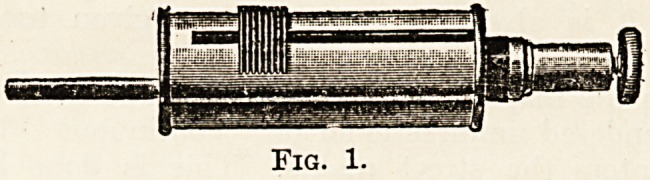


**Fig. 2. f2:**
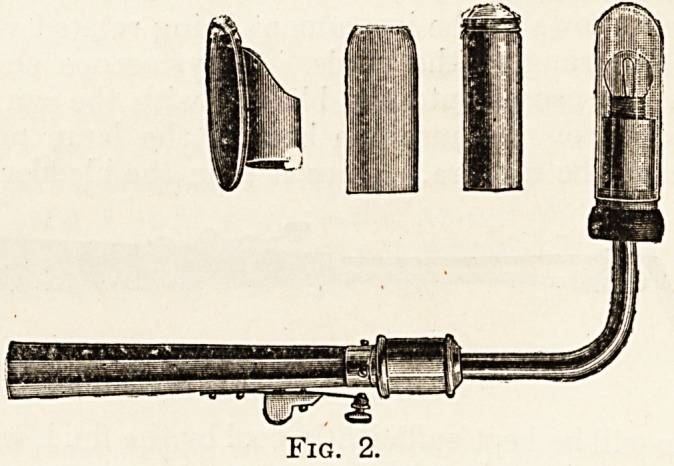


**Fig. 3. f3:**